# 15例儿童非免疫缺陷原发中枢神经系统淋巴瘤的临床研究

**DOI:** 10.3760/cma.j.cn121090-20230904-00102

**Published:** 2024-02

**Authors:** 慧霞 高, 宁宁 张, 春菊 周, 玲 金, 菁 杨, 爽 黄, 梦 张, 楠 李, 永红 张, 彦龙 段

**Affiliations:** 1 国家儿童医学中心，首都医科大学附属北京儿童医院儿童肿瘤中心肿瘤内科，儿童血液病与肿瘤分子分型北京市重点实验室，儿童肿瘤国家临床重点专科，儿科重大疾病研究教育部重点实验室，北京 100045 Medical Oncology Department, Pediatric Oncology Center, Beijing Children's Hospital, Capital Medical University, National Center for Children's Health, Beijing Key Laboratory of Pediatric Hematology Oncology, National Key Clinical Discipline of Pediatric Oncology, Key Laboratory of Major Diseases in Children, Ministry of Education, Beijing 100045, China; 2 首都医科大学附属北京儿童医院影像中心，北京 100045 Department of Imaging, Beijing Children's Hospital, Capital Medical University, Beijing 100045, China; 3 首都医科大学附属北京儿童医院病理科，北京 100045 Department of Pathology, Beijing Children's Hospital, Capital Medical University, Beijing 100045, China

## Abstract

回顾性分析首都医科大学附属北京儿童医院2013年5月至2023年5月收治年龄≤18岁的15例非免疫缺陷原发中枢神经系统淋巴瘤（PCNSL）患儿的临床资料，总结临床特点以及基于病理组织亚型的、以大剂量甲氨蝶呤（HD-MTX）为基础的化疗方案的治疗效果。男女比例2.7∶1，中位确诊年龄7.2（2.3～16.4）岁，临床首发症状以颅高压伴颅神经受损为主，影像学以多发病灶为主。免疫功能正常的儿童PCNSL发病率极低，以HD-MTX为基础的化疗整体预后良好，部分病情评估稳定者可减量维持或不予维持治疗。初治以非HD-MTX为主的化疗，病情进展或复发患儿再次予HD-MTX为基础的化疗仍有效。

## Abstract (en)

Clinical data of 15 primary central nervous system lymphoma (PCNSL) children aged ≤18 years admitted to our hospital between May 2013 to May 2023 were retrospectively analyzed. Our goal was to summarize the clinical features of children and investigate the therapeutic effect of a high-dose methotrexate (HD-MTX) based chemotherapy regimen on this disease. The male-to-female ratio was 2.7∶1, and the median age was 7.2 (2.3–16.4) years at diagnosis. The initial clinical symptoms were primarily cranial hypertension, with imaging findings revealing multiple lesions. Pediatric PCNSL with normal immune function has a favorable prognosis with HD-MTX-based chemotherapy. Patients with a stable disease can be treated with minimal or no maintenance. HD-MTX-based chemotherapy remains effective when the disease progresses or recurs after an initial course of non-HD-MTX-based chemotherapy.

原发性中枢神经系统淋巴瘤（Primary central nervous system lymphoma, PCNSL）原发于中枢神经系统，可累及脑实质、脊髓、颅神经、眼和软脑膜，而无全身播散证据[1]。本病在儿童和青少年中是一种罕见的结外非霍奇金淋巴瘤（NHL）亚型，好发于免疫缺陷患者[2]–[3]。免疫功能正常的儿童PCNSL罕见报道，确切发病率不详，据国际柏林-法兰克福-蒙斯特（Berlin-Frankfurt-Munster，BFM）组织的一项多中心研究统计，欧洲19岁以下无HIV感染的儿童年发病率仅0.43％（10/2 311）[3]。因儿童PCNSL发病罕见，其确切的生存率和预后尚不清楚。标准诱导方案和巩固治疗时长尚未明确，近年来，以大剂量甲氨蝶呤（HD-MTX）（5～8 g/m^2^）为基础的化疗使得患儿5年生存率有所提高[4]–[5]。本研究回顾分析单中心近10年收治的15例非免疫缺陷PCNSL患儿的临床数据，以期进一步认识本病的临床特征、治疗现状及预后。

## 病例与方法

1. 病例：收集首都医科大学附属北京儿童医院2013年5月至2023年5月收治的15例免疫功能正常的PCNSL患儿的临床资料进行回顾性研究，13例患儿通过立体定向穿刺活检或颅内占位切除获取病变组织标本，经病理形态及免疫组织化学确诊，2例经脑脊液（CSF）流式细胞术确诊。纳入标准：①病理确诊为NHL，且符合2008年WHO造血和淋巴组织肿瘤分类标准[6]；②初诊筛查体液及细胞免疫功能正常，既往不伴随其他恶性肿瘤或免疫缺陷病；③全身正电子发射断层扫描（PET-CT）及超声排除其他原发部位淋巴瘤累及中枢神经系统；④有完整的治疗和随访资料。

2. 收集临床资料：回顾性分析患儿病历资料，进一步收集临床特征、实验室及影像学检查、诊疗经过及随访结局。

3. 治疗方案：基于病理组织类型，采用以HD-MTX（3～8 g/m^2^）为基础的化疗方案：①侵袭性成熟B细胞型PCNSL患儿采用我院改良的LMB89方案中枢侵犯组（C2组）[7]或D组方案［本研究中2例间变大B细胞淋巴瘤（ALCL）采用］，即以HD-MTX（5～8 g/m^2^）和大剂量阿糖胞苷（HD-Ara-C）（3 g/m^2^）为骨干药物，联合鞘内注射治疗。视病情评估给予全程或减量或免维持治疗。第一个疗程COP方案7 d，之后每疗程化疗间隔16～21 d，总治疗时长4～6个月。②B-淋巴母细胞型PCNSL患儿采用我院改良的BFM-95方案，包括VDLP+CAM诱导缓解，4个疗程HD-MTX（3 g/m^2^）或6个疗程高危方案的缓解后巩固治疗，VDLD+CAM延迟强化治疗，6-MP+MTX维持治疗等，总疗程约24个月。

4. 疗效评估：评估内容包括脏器功能及病毒感染情况，影像学（包括CT、MRI）评估颅内瘤灶变化，并定期完善全身PET-CT、CSF及眼科检查等。LMB89方案评估节点包括预治疗（COP方案）后第7天，中期评估［第4疗程CYVE1（HD-Ara-C 3 g/m^2^、依托泊苷）后］和停止化疗后；BFM-95方案评估节点包括诱导阶段（第8、15和33天）、中期评估［CAM2（环磷酰胺、巯嘌呤、Ara-C及三联鞘内注射）化疗后］、延迟强化前期及化疗维持期。采用2005年国际PCNSL协作组共识指南[8]评价标准，疗效评价包括完全缓解（CR）、部分缓解（PR）、疾病进展（PD）、疾病稳定（SD）。

5. 随访：随访截止日期为2023年5月31日。患儿化疗结束后定期门诊复查随访，停止化疗2年内每3个月复查1次，之后每半年复查1次，视患儿病情监测血常规，评估脏器和免疫功能及瘤灶部位影像学等。主要随访终点为初次无进展生存（PFS1）期，即从确诊至初次病情进展的时间；次要随访终点为无事件生存（EFS）期和总生存（OS）期。EFS期定义为从诊断之日起至首次事件（包括复发、进展和死亡）发生之日。OS期定义为从诊断到任何原因引起死亡的时间。

6. 统计学处理：采用SPSS 及Graphpad prism软件进行统计学分析，预后分析采用Kaplan-Meier法，以*P* ≤ 0.05为差异有统计学意义。

## 结果

1. 基本资料：男女比例2.7∶1，中位诊断年龄7.2（2.3～16.4）岁。中位确诊前病程40（14～240） d，4例（26.7％）在症状出现4周内确诊。临床首发症状为头晕、头痛6例（伴复视2例，伴抽搐1例），眼部包块3例，双下肢无力或行动受限3例，其余头部包块、鼻腔肿物和背痛起病各1例。影像学检查结果显示单发病灶4例，多发病灶11例，病灶最常累及脑实质（额/颞叶多见）和脑室（Ⅳ脑室多见），脊髓和眼眶发病相当。13例经脑内瘤灶立体定向活检确诊，2例经CSF流式细胞术确诊。病理分型：ALCL 2例，高级别B细胞淋巴瘤3例，伯基特淋巴瘤3例，B-淋巴母细胞淋巴瘤7例。超声及PET-CT评估未见其他躯体广泛转移，均无巨大瘤灶；均未见骨髓转移，免疫功能指标均正常。HIV、EB病毒和乙型肝炎病毒检测结果均为阴性。患儿临床特征详见表1。

**表1:** 15例非免疫缺陷原发中枢神经系统淋巴瘤患儿临床特征

例号	年龄（年）	性别	确诊方式	诊断	病灶位置	起病表现	确诊前病程（d）	CSF cell/pro（/L；mg/L）	眼内累及	融合基因	复发/进展	WBRT	合并症
1	4	女	活检	ALCL	脊髓	背痛、后背弯曲	60	双阴性	N	NPM::ALK	N	N	肿瘤溶解综合征；BM抑制；黏膜炎
2	7	女	活检	ALCL	左额叶/右颞叶	发热、鼻腔肿物	180	双阴性	N	NPM::ALK	N	N	肿瘤溶解综合征；BM抑制；颅内出血；黏膜炎
3	13	男	切除+活检	BL	Ⅳ脑室	头晕/痛、耳鸣	120	双阴性	N	c-MYC	N	N	BM抑制；黏膜炎
4	14	男	切除+活检	BL	Ⅳ脑室、小脑	头晕/痛	30	双阴性	N	N	N	N	BM抑制；黏膜炎
5	14	男	切除+活检	BL	左额/颞叶、左眼	头痛、抽搐	90	双阴性	Y	N	Y	N	BM抑制
6	5	男	CSF-FCM	HGBCL	双侧额叶、双侧视神经	头痛、复视	40	432×10^6^；1302	Y	N	N	N	肿瘤溶解综合征；BM抑制；黏膜炎
7	16	男	切除+活检	HGBCL	Ⅱ/Ⅲ脑室、左额/双颞叶	头痛	14	双阴性	N	N	Y	Y	BM抑制；败血症；抽搐；静脉血栓
8	7	男	切除+活检	HGBCL	右眼眶、右额/颞叶	右眼眶肿物	60	双阴性	Y	N	N	N	BM抑制
9	3	女	切除+活检	B-LBL	小脑	行走不稳	60	双阴性	N	N	N	N	BM抑制；慢性硬膜下血肿
10	2	男	活检	B-LBL	左额叶	头部包块	30	46×10^6^；阴性	N	E2A::PBX1	Y	N	肿瘤溶解综合征；BM抑制；肝损伤，胆汁淤积
11	5	男	活检	B-LBL	脊髓、右眼	右眼结膜肿物	240	25×10^6^；阴性	Y	N	N	N	肿瘤溶解综合征；BM抑制；肝损伤，胆汁淤积；黏膜炎
12	8	男	CSF-FCM	B-LBL	脊髓、脑膜	头痛、复视	21	78×10^6^；阴性	N	N	N	N	肿瘤溶解综合征；BM抑制；肝损伤，胆汁淤积
13	6	女	切除+活检	B-LBL	右眼眶	右眼包块	30	双阴性	Y	N	Y	N	BM抑制；败血症；黏膜炎
14	2	男	切除+活检	B-LBL	脊髓	双下肢无力	20	双阴性	N	N	N	N	BM抑制
15	6	男	切除+活检	B-LBL	脊髓	腰骶痛、活动受限	40	双阴性	N	N	N	N	BM抑制

注 ALCL：间变大B细胞淋巴瘤；B-LBL：B-淋巴母细胞淋巴瘤；BL：伯基特淋巴瘤；HGBCL：高级别B细胞淋巴瘤；CSF cell：脑脊液细胞数；CSF pro：脑脊液蛋白定量；CSF-FCM：脑脊液流式细胞术；WBRT：全颅放疗；BM：骨髓；Y：有；N：无

2. 治疗及转归：15例患儿均无失访或治疗相关死亡，中位随访时间为33.5（12～112）个月。10例随访超过2年，2年EFS率为98.3％，7例随访超过5年，5年OS率为100％。8例原发中枢神经系统侵袭性淋巴瘤患儿予改良的LMB89方案化疗：3例予4个周期维持治疗，3例减维持（仅予1个周期）治疗，2例未予维持治疗。除2例（例5、例7）首次外院接受非HD-MTX为主的化疗方案，3个月内评估均出现病情进展，收治我院后重新予HD-MTX为主的LMB89方案化疗：例5随访近10年，持续CR；例7中期及维持期评估均CR，但共济失调及尿便排出障碍症状缓解不明显，接受了全颅放射（总放疗量23.4 Gy），前述症状获得缓解，截至随访日无病情复发或进展。其余6例原发中枢神经系统侵袭性淋巴瘤患儿均于我院首次接受LMB89方案化疗，目前随访超过2年，均无事件生存。7例B-淋巴母细胞型PCNSL患儿于我院接受BFM-95方案：5例治疗及随访评估均持续CR；2例早期评估PR，升级高危方案后1例达CR，1例仍在治疗观察中。

3. 治疗相关合并症：6例（40.0％）诊断时及COP方案预治疗阶段存在肿瘤溶解综合征，1例COP方案后合并颅内出血，1例合并慢性硬膜下血肿，2例合并败血症，3例合并肝损害并胆汁淤积，部分患儿存在消化道黏膜炎，全部患儿在诱导及巩固化疗阶段合并骨髓抑制。无患儿死于肿瘤溶解综合征及上述相关合并症，至随访截止日均无神经系统后遗症状或第二肿瘤综合征。患儿的一线治疗及随访评价见表2。

**表2:** 15例非免疫缺陷原发中枢神经系统淋巴瘤患儿的一线治疗方案及随访评价

例号	初治方案a/b/*	早期评估	中期评估	维持治疗疗程	维持期评估	PFS1期（月）	EFS期（月）	OS期（月）	随访评估
1	a	CR	CR	1	CR	114	114	114	NED
2	a	CR	CR	1	CR	80	80	80	NED
3	a	CR	CR	1	CR	81	81	81	NED
4	a	CR	CR	4	CR	60	60	60	NED
5	*	PD	CR	4	CR	3	109	112	NED
6	a	CR	CR	N	CR	24	24	24	NED
7	*	PD	CR	4	CR	3	9	12	NE
8	a	CR	CR	N	CR	26	26	26	NED
9	b	CR	CR	Y	CR	41	41	41	NED
10	b	PR	PR	−	−	1	16	17	NE
11	b	CR	CR	−	−	15	15	15	NE
12	b	CR	CR	−	−	16	16	16	NE
13	b	PR	CR	−	−	1	11	12	NE
14	b	CR	CR	Y	CR	112	112	112	NED
15	b	CR	CR	Y	CR	102	102	102	NED

注 方案a：改良的LMB89方案；方案b：改良的BFM95方案；*：外院非方案a 或b； NED：无疾病证据； NE：未评估到；PFS1：初次无进展生存；EFS：无事件生存；OS：总生存；CR：完全缓解；PD：疾病进展；PR：部分缓解；N：未予维持治疗；Y：予以维持治疗，初治采用改良的BFM95方案未关注疗程数；−：尚未进入维持期/未进行评估

## 讨论

免疫功能正常的儿童PCNSL不同于成人，发病率极低，本研究发现采用以HD-MTX为骨干药物、针对组织病理学亚型的化疗可获得良好预后。本组回顾性研究是迄今为止最大的单中心儿童和青少年PCNSL病例系列。本中心10年儿童NHL病例中PCNSL发病率约为1％（15/1 500），略低于日本先前报道的比例（1.5％，9/596例）[9]。疾病的罕见性使得实际临床中难以对儿童患者进行大规模前瞻性治疗研究，其确切的生存率和预后尚不清楚。目前最大的儿童PCNSL系列研究（75例）[3]报道中，除外存在先天或继发免疫缺陷的病例，56例免疫功能正常的PCNSL患儿5年EFS率为（77±6）％。国内成人神经外科报道[10]了11例儿童PCNSL患者，其平均生存时间为44.4个月，5例死亡患儿平均生存时间仅8.8个月。本组研究中有10例患儿随访超过2年（EFS率为98.3％），7例随访超过5年（OS率为100％），且所有患儿仍在治疗和随访中，我们呼吁尽量将儿童PCNSL患者收治于专科儿童医院并予规范的以化疗为主导的治疗方案。

PCNSL的确诊依赖病理检查，立体定向活检是获取组织病理诊断的主要方法。儿童PCNSL患者男性更常见，本组患儿男女比例2.7∶1，发病年龄与既往报道相符[11]。颅脑MRI是诊断本病的主要影像学方式，病灶可单发或呈多灶性，病灶信号多数较均等，瘤内出血和坏死少见，典型表现为T2加权相呈现等或低信号[12]。本组头颅MRI示最常累及部位为脑实质（额/颞叶多见）、脑室（Ⅳ脑室多见），脊髓和眼眶发病相当，部分病例累及小脑，这也与典型的临床表现[12]–[13]包括颅高压症状（头痛、呕吐）伴局灶性颅神经功能受损症状（复视、面神经麻痹、构音障碍）、小脑症状（共济失调）及眼部症状（视物模糊、眼球震颤、眼球突出等）相符。癫痫发作和偏瘫也很常见，累及脊髓者，其临床特点类似于其他髓内肿瘤。本组患儿中位确诊前病程40（14～240） d，4例（26.7％）在症状出现的4周内确诊。由于本病临床症状多样且不具特异性，给实际临床诊疗带来挑战，对无发热等感染征象、兼有颅内或脊髓病变、抗感染疗效不佳且不能解释疾病全貌时需警惕本病可能。

儿童PCNSL患者的预后比大多数成人患者好[14]，这可能得益于儿童患者比成人能耐受更高剂量的MTX。由于PCNSL具有弥漫性浸润特点，单纯手术切除病灶仅能使中位生存期延长1～4个月[12]。二十年来，以HD-MTX（5～8 g/m^2^）和HD-Ara-C（3 g/m^2^）为主要药物的LMB89方案一直是儿童和青少年成熟B-NHL的样板方案[15]，诸多回顾性病例报道均提示含有HD-MTX的系统性多药联合化疗对儿童PCNSL有效[11],[16]–[17]，可使患儿获得良好的生存率[12]。本组研究中，基于组织病理分型，予8例成熟侵袭性淋巴瘤患儿改良的LMB89方案（2例ALCL患儿予LMB89-D组方案），其中3例减维持治疗，2例未予维持治疗。除外2例（例5、例7）首次外院接受非HD-MTX为主的化疗（外院化疗方案中MTX为3 g/m^2^），并于3个月内出现病情进展，其余6例于我院初诊初治患儿均获得持续缓解。此2例病情进展患儿收入我院后，经复核组织病理标本确认诊断无误后，重新予HD-MTX为主的LMB89方案化疗。本组研究提示以HD-MTX为主的系统性多药联合化疗可能是控制儿童PCNSL病情的有效选择。特别指出，首次以非HD-MTX为主导药物化疗并出现疾病进展或复发者，可再次尝试以HD-MTX为基础的化疗，本组研究中的2例病情进展患儿获得良好的化疗反应和PFS。此外，近2年来，本单中心尝试予病情评估稳定的患儿减量维持或不再给予维持治疗，本组中3例患儿仅予1个周期维持治疗，2例未予维持方案，目前随访超过2年，均获得持续性CR。这也提示非免疫缺陷PCNSL患儿早期接受以HD-MTX为基础的规范治疗，后续病情评估稳定的情况下可尝试减量维持甚至不给予维持治疗，总体预后良好。

本研究是回顾性描述研究，可能存在一定的报道偏差。本研究纳入分析的患儿数量有限，不同组织类型的病例数较少，需要更长的随访时间来描述长期存活或晚期复发患儿的数量。但考虑到儿童PCNSL发病的罕见性，迫切需要加强对本病治疗规范和临床疗效的进一步认识。

综上，以HD-MTX为基础的多药联合化疗对免疫功能正常的儿童PCNSL总体疗效良好，无需全颅放疗。部分病情评估稳定者可尝试减量维持甚至不予维持治疗，初治以非HD-MTX为主的化疗方案，病情进展或复发患儿再次给予HD-MTX为基础的化疗可能是控制病情的有效选择。
